# Breaking the Hybrid–Species Barrier

**DOI:** 10.1371/journal.pbio.1001201

**Published:** 2011-11-15

**Authors:** Robert Shields

**Affiliations:** Freelance Science Writer, Cambridge, United Kingdom

**Figure:**
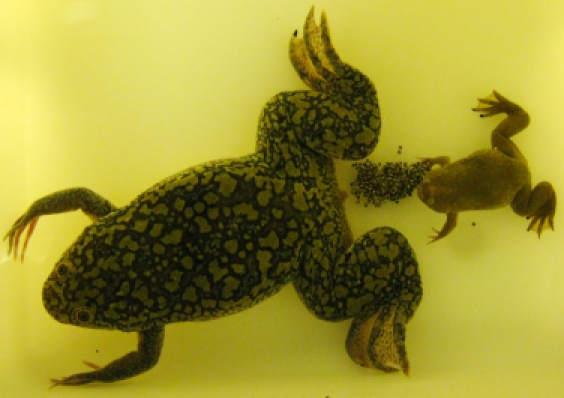
A *Xenopus tropicalis* male seems interested in making use of eggs laid by its 60-million-year relative sister *Xenopus laevis*.

It's been more than 50 years since John Gurdon first placed a nucleus from a differentiated cell of a *Xenopus laevis* tadpole into an enucleated *Xenopus* egg and regenerated a complete animal. This demonstrated two important facts: the somatic cell nucleus contains the complete genetic information needed for the production of the organism, and nuclei of differentiated cells can be reprogrammed into an embryonic state by the egg cytoplasm. Nowadays, this remarkable feat of reprogramming to generate a whole animal is almost commonplace, with Dolly the sheep being perhaps the most famous example. We now know that in some species reprogramming of nuclei in differentiated tissue can be achieved (albeit at low efficiency) by as few as four protein factors. And we know that the egg cytoplasm of some mammals can reprogram the mature nuclei of closely related species to the extent that live animals can be obtained (in the case of cow and gaur, for instance), while reprogramming of cells from more widely diverged species is only partially successful. What we don't know is why—and at what stage—divergent species reprogramming fails?

In research reported in this issue of *PLoS Biology*, Narbonne, Simpson, and Gurdon tackled this problem using two frog species—*X. laevis* and *Xenopus tropicalis*—separated by more than 60 million years of evolution. They show that the failure to reprogram distantly related species may arise from a small suite of factors rather than from a systemic failure, suggesting a strategy for overcoming this longstanding developmental mystery.

To approach this problem, the researchers inactivated the resident nucleus of eggs with ultraviolet light and fertilized them with sperm from one or the other species. When sperm and egg were from the same species, viable haploid tadpoles developed, showing that sperm nuclei can be correctly reprogrammed by the egg cytoplasm. In contrast, when sperm and egg came from the diverged species—that is, when inactivated *X. laevis* eggs were fertilized with *X. tropicalis* nuclei—they failed to develop properly. Despite active cell division, the embryos stalled around a stage known as gastrulation.

This failure could result from some defect in nuclear reprogramming, or it might result from an incompatibility between the two species. For example, the “foreign” nucleus could interfere with the normal function of the host cytoplasm.

An important stage in somatic nuclear reprogramming is embryonic gene activation (EGA), when new embryo-specific gene transcripts and new embryonic proteins from the mature nucleus are produced. The researchers found, at least in a limited sampling, that this stage seemed to proceed normally for the foreign nucleus and host egg species (although the authors acknowledge that a wider sampling might have revealed differences). Another obvious question is whether the foreign nucleus can cooperate with the mitochondria of the foreign cytoplasm. Mitochondria produce the energy currency of the cell—ATP—and have their own genomes, which produce some of the proteins needed for ATP generation; the nucleus encodes the remaining proteins. It's possible that millions of years of evolution and species divergence produced an incompatibility between the host mitochondria and the new nucleus, resulting in energy deficiency. Again, this did not seem to be the case (although ATP is not the only mitochondrial product that requires nucleus and cytoplasm cooperation; precursors for fats are also produced in the mitochondria).

The researchers found another potential point of developmental failure for the cross-species hybrids: the hybrid embryos didn't seem to organize properly to form the long axis, which is critical for normal development. This could result from a failure to produce the signaling molecules that normally produce cell elongation or from a failure to read such signals. The answer seems to be a bit of both, as explants of the hybrid embryos elongate better when conjoined to parts of non-hybrid embryos, and non-hybrids do not elongate as well when attached to hybrids. Thus, a failure to produce and respond to elongation-signaling molecules offers a partial explanation. The authors noted that the hybrid embryos seemed to be relatively short of Xbra, a protein that regulates gene expression and is known to be important for convergence extension. By up-regulating Xbra expression in hybrid embryos, they could improve embryo elongation.

Taken altogether, these results suggest that divergent species hybrids may fail as a result of a limited number of deficiencies—which, on a positive note, could conceivably be overcome by modulation of relatively few factors—rather than arising from gross failures of universal processes such as embryonic gene expression or energy metabolism.

Why should we care about hybridization between divergent species? One day, it might be possible to enlist hybridization to develop a surrogate breeding system using differentiated cells of endangered or even extinct animals and eggs from a more readily available animal. Somatic cell nuclear reprogramming and development in a surrogate cytoplasm might also provide a better and more acceptable source of embryonic stem cells for therapy than current methods. But at the most basic level, understanding the mechanisms that lead to reproductive isolation helps us understand how species diverge over evolutionary time.

**Narbonne P, Simpson DE, Gurdon JB (2011) Deficient Induction Response in a** ***Xenopus*** **Nucleocytoplasmic Hybrid. doi:10.1371/journal. pbio.1001197**

